# Exploring Differences in the Role of Hospitalization on Weight Gain Based on Treatment Type From Randomized Clinical Trials for Adolescent Anorexia Nervosa

**DOI:** 10.3389/fpsyt.2020.609675

**Published:** 2020-11-12

**Authors:** Nandini Datta, Brittany E. Matheson, Daniel Le Grange, Harry A. Brandt, Blake Woodside, Katherine A. Halmi, Denise E. Wilfley, James D. Lock

**Affiliations:** ^1^Department of Psychiatry and Behavioral Sciences, Stanford University School of Medicine, Stanford, CA, United States; ^2^Department of Psychiatry and Behavioral Sciences, University of California, San Francisco, San Francisco, CA, United States; ^3^Department of Psychiatry and Behavioral Neurosciences, The University of Chicago, Chicago, IL, United States; ^4^St. Joseph Medical Center, University of Maryland, Towson, MD, United States; ^5^Eastern Region Eating Recovery Center, Towson, MD, United States; ^6^Program for Eating Disorders, Toronto General Hospital, Toronto, ON, Canada; ^7^Department of Psychiatry, University of Toronto, Toronto, ON, Canada; ^8^Department of Psychiatry, Weill Cornell Medical College, New York, NY, United States; ^9^Department of Psychiatry, Washington University in St. Louis, St. Louis, MO, United States

**Keywords:** anorexia, hospitalization, inpatient, weight gain, treatment outcome, adolescent

## Abstract

**Background:** This study explores the impact of weight gain during medical stabilization hospitalization on weight outcomes between three outpatient treatments for adolescent anorexia nervosa (AN): Adolescent Focused Therapy (AFT), Systemic Family Therapy (SyFT), and Family Based Treatment (FBT).

**Methods:** A secondary analysis of weight gain data (*N* = 215) of adolescents (12–18 years) meeting DSM-IV criteria for AN (exclusive of amenorrhea criteria) who participated in two randomized clinical trials (RCTs) was conducted. Main outcomes examined were changes in weight restoration (≥95% expected body weight or EBW) and differences in weight change attributable to hospital weight gain.

**Results:** Weight gain resulting from hospitalizations did not substantially change weight recovery rates. Hospital weight gain contributed most to overall treatment weight gain in AFT compared to FBT and SyFT.

**Conclusion:** Brief medical stabilization weight gain does not contribute substantially to weight recovery in adolescents with AN who participated in RCTs.

## Introduction

Medical hospitalization to address the physiological effects of starvation and related maintaining behaviors of Anorexia Nervosa (AN) plays an important role when treating adolescents with AN. However, the extent to which hospitalization contributes to weight restoration is unclear. The few available studies suggest limited impact (1, 2). Madden et al. (3) found no benefit of longer term hospitalization aimed at achieving higher weights for patients receiving Family-based Treatment (FBT) upon discharge, suggesting that limited hospital stays were sufficient for these patients. Additionally, prior studies found that hospitalization rates differ between outpatient treatments for adolescents with AN, leading to significant differences in cost-effectiveness between treatment types (4, 5). However, it is unknown whether hospitalization during evidence-based outpatient treatments for adolescent AN differentially contributes to overall weight outcomes depending on treatment type. Thus, the purpose of this retrospective exploratory study is to examine the relative impact of hospital weight gain on treatment outcomes [i.e., weight restoration: ≥95% expected body weight (EBW)] (6, 7) in the context of three outpatient treatments: FBT (8), Adolescent Focused Therapy (AFT) (9), and Systemic Family Therapy (SyFT) (10), employed in two randomized clinical trials (RCTs) for adolescents with AN.

While hospitalization for psychiatric and behavioral treatment of adolescent AN is a potentially important component of care and outcome, the current study focuses on the role of brief medical hospitalization used to treat medically unstable adolescents with AN in studies of outpatient psychosocial interventions for this disorder. These hospitalizations are unplanned and occur in response to medical instability. Medical instability results from physiological impacts of behaviors that maintain AN and can result in bradycardia, hypotension, and orthostatic hypotension (11). In addition, starvation and rapid re-feeding can result in blood chemistry changes leading to potentially lethal re-feeding syndrome (12). Treatments during medical hospitalization vary, but in general these admissions focus on promoting safe but rapid weight gain through close meal monitoring, limited activity, focused psychological support for patients and parents, and preparation for discharge to outpatient care (13). Lengths of stay for medical hospitalization vary but are usually between 1 and 3 weeks for adolescents in the US and Canada (14, 15).

There are few RCTs for adolescent AN; two relatively large studies examined individual therapy aimed at promoting adolescent development in the context of AN (AFT), systemic family therapy (SyFT) aimed at improving family communication and process in the context of AN, and family-based treatment (FBT) aimed at parental behavioral management of weight gain and adolescent development (4, 5). Results of these RCTs found that FBT had higher rates of recovery than AFT at follow-up and was more cost-effective than SyFT.

While previous studies demonstrate that FBT generally uses less medical hospitalization than AFT and SyFT (4, 5), the impact of hospitalization on weight gain itself is unaccounted for in the outcomes. Thus, weight gain during hospital admissions might vary between treatment types and contribute differentially to weight restoration. Based on the previous studies we expect that when looking at the entire sample (both hospitalized and non-hospitalized participants), hospital weight gain will likely have minimal effects on weight recovery rates, or remission, regardless of treatment type. When looking at the impact of hospitalization weight gain on end-of-treatment (EOT) weight outcomes, we predict differing effects of hospitalization depending on treatment type. Specifically, we anticipate greater overall impacts on weight at EOT for participants receiving individual treatment (AFT) compared to family treatment (FBT or SyFT). This hypothesis is rooted in prior literature (4), reporting that in AFT, alliance building and relatedly weight progress takes longer than in FBT, resulting in a prolonged period of time during which the adolescent is more medically vulnerable. Hospitalization for adolescents receiving AFT may serve as a “safety net” while they develop the motivation and skills to change behaviors independently. These hypotheses are exploratory due to the preliminary nature of this study. Results stand to inform future systematic studies designed to investigate the role of hospitalization on weight outcomes in adolescents receiving evidence-based treatments for AN.

## Materials and Methods

Data used in this study were collected from two RCTs of adolescents (ages 12–18) who met DSM-IV criteria for AN (exclusive of amenorrhea criteria). The first clinical trial randomized participants (*N* = 121) to receive either FBT or AFT in a two-site study (4); the current data are only those recruited at the Stanford site (*N* = 60), as The University of Chicago did not have a dedicated inpatient unit and participants were admitted, when appropriate, to facilities outside of the university. The second clinical trial randomized participants (*N* = 158) to receive FBT or SyFT in a 6-site study (5). For this paper, the FBT samples from the two RCTs were combined. The studies were reviewed and approved by their respective Institutional Review Boards and all participants provided written informed consent or assent.

The criteria for hospitalization at the time of the study followed the guidelines of the American Academy of Pediatrics (16) and the Society for Adolescent Medicine (17) for medical hospitalization of adolescents with AN: heartrate <45 beats-per-minute, orthostatic blood pressure changes >35 points, gastrointestinal bleeding, dizziness, syncope, EBW <75%, body temperature below 36°C, electrolyte abnormalities, and/or prolonged QTc. Using these criteria, a total of 59 participants (27%) were hospitalized for medical reasons during the treatment period of the two studies: AFT = 48% (16/33); SyFT = 22% (17/79); FBT = 19% (20/106).

Demographic characteristics of the hospitalized sample can be found in Table 1. The outcome variables of interest were: (1) Hospitalization days and number of admissions for medical stabilization by treatment type; (2) Total hospital weight gain; (3) Treatment weight gain; (4) Change in EOT weight outcomes (defined as EBW percent for age, height, and sex, using CDC norms) from baseline to EOT; (5) Timing of hospitalization (weeks in treatment until first hospitalization). Weight was measured and is reported in kilograms.

**Table 1:** Demographics by treatment type.

	***AFT (N* = 33)**	***SyFT (N* = 79)**	***FBT (N* = 106)**
Age M (SD)	14.7 (1.3)	15.0 (2.4)	14.4 (1.7)
**Sex (%)**			
Male	3%	7.6%	13.2%
Female	97%	91.1%	86.8%
**Race (%)**			
Asian	18.2%	5.1%	10.4%
Black or African American	3%	–	–
White	72.7%	92.4%	82.1%
More than one race	6.1%%	2.5%	7.5%
**Ethnicity (%)**			
Hispanic/latino	6.1%	12.7%	11.3%
Not hispanic/latino	93.9%	87.3%	88.7%
Baseline % EBW M (SD)	80.0 (3.8)	81.8 (3.7)	82.1 (3.6)
Medications at baseline (%)	6%	37.9%	24.5%

The effect of hospitalization on weight outcomes according to treatment allocation was calculated by subtracting the mean hospital weight gain from the mean weight gain at EOT for each individual participant and from this, calculating a mean difference score for weight. This new “adjusted” weight was then used to re-calculate percent EBW for age, height and sex using CDC norms, producing an “adjusted EBW” for each participant, accounting for hospitalization weight gain. Weight recovery or remission was defined as ≥95% EBW, consistent with prior research demonstrating this criterion as the best predictor for long-term recovery (6, 7). The entire sample, including those not hospitalized, was assessed to calculate differences in number of people meeting weight recovery after accounting for hospital weight gain.

Timing (by weeks of treatment) of hospitalizations was determined by average number of weeks in treatment to first hospitalization by group and number of weeks in treatment to each admission thereafter (if there were multiple admissions). Frequency of hospitalizations and multiple admissions were not controlled for in the current analyses.

Data were analyzed using SPSS version 26.0. Analyses involving the subset of participants who were hospitalized were not normally distributed and had unequal cell sizes, thus non-parametric statistics and *post-hoc* analyses were utilized. All measures of central tendency reported are medians (Mdn) with Interquartile Ranges (IQR) or frequencies (count). Due to the exploratory nature of this study, we report effect sizes (ES) for comparisons, specifically, success rate differences (SRD).

## Results

For those hospitalized during the study, the median number of hospitalized days in SyFT was 23 (IQR = 15–31), compared to a median of 6 days in AFT (IQR = 4–14) and 9.5 days in FBT (IQR = 5–16.8).

### Weight Remission

Medical hospitalization had little effect on the number of participants who were weight recovered at EOT. When accounting for weight gained during hospitalization, the recovery rates in each group were: FBT = 44/106 or 41.5% (unchanged); SyFT = 21/79 or 26.6% (vs. 27.8% when including hospitalization weight gain); AFT = 6/33 or 18.2% (vs. 21.2% when including hospitalization weight gain).

### Weight Change

Hospitalization impact on weight outcomes for the entire sample differed depending on the type of outpatient treatment: hospitalization contributed most to EOT weight outcomes in AFT where 7.2% of EBW was attributable to hospital weight gain (Table 2). For SyFT and FBT, hospital weight gain did not contribute to EBW at EOT. The differences in percent EBW at EOT after removing hospital weight gain (i.e., “adjusted EBW”) differed between treatment arms with large effect sizes, with AFT < SyFT: Mann-Whitney *U* = 773.5; SRD = 0.40 and AFT < FBT: Mann-Whitney *U* = 901.5; SRD = 0.48.

**Table 2:** Hospitalization outcomes^†^.

**Entire sample**	***AFT (N* = 33)**	***SyFT (N* = 79)**	***FBT (N* = 106)**	**SRD^**‡**^**
% of total sample hospitalized	49%	22%	19%	
Number of admissions	42	19	26	
Number of patients with repeat admissions	8 of 33	5 of 79	5 of 106	
Weight gained in treatment (kg)	5 (1.6–7.9)	4.9 (2.3–8.7)	5.9 (3.5–10.2)	
% EBW EOT	89 (79.6–93.9)	89 (84.1–95.3)	92.6 (86.2–98.1)	
% EBW “adjusted” (hospital weight gain removed)	81.8 (75.9–92.9)	88.9 (84.1–95.4)	92.6 (86–96.9)	AFT < SyFT: 0.40
				AFT < FBT: 0.48
% change in EBW attributable to hospitalization	7.2%	0%	0%	
Number of patients ≥95% EBW	7	22	44	
% of total sample weight restored	21.2%	27.8%	41.5%	
Number of patients ≥95% EBW (after removing hospital weight gain)	6 (vs. 7)	21 (vs. 22)	44 (vs. 44)	
Adjusted % of total sample weight restored	18.2%	26.6%	41.5%	
**Hospitalized Sample**	**AFT (*****N*** **=** **16)**	**SyFT (*****N*** **=** **17)**	**FBT (*****N*** **=** **20)**	**SRD**^**‡**^
Median number of days hospitalized	6 (4–14)	23 (15–31)	9.5 (5–16.8)	SFT>FBT: 0.54
				SFT>AFT: 0.69
Weight gained in treatment (kg)	3.9 (2–5.7)	3.5 (2–5)	3.1 (1.5–6.6)	
% EBW EOT	88 (77.8–94.9)	83.7 (80.1–94.2)	92 (79.7–101.2)	
% EBW “adjusted” (hospital weight gain removed)	78 (71.3–89.74)	78.9 (73.7–92.4)	92 (73.3–96.7)	

† *All statistics are reported medians (Mdn) and interquartile range (IQR) unless otherwise specified*.

‡ *Effect size used is success rate difference (SRD) given the use of pairwise comparisons of treatments with unequal variances. Here, SRD = 0.1, 0.3, 0.4 can be interpreted as small, medium, and large effect sizes, respectively (18)*.

### Timing of Hospitalization

For FBT and SyFT more than half of all hospitalizations occurred within the first 5 weeks of treatment. In FBT, 54% of the sample was hospitalized within the first 5 weeks (*n* = 14/26) and in SyFT, 57% of the sample was hospitalized within the first 5 weeks (*n* = 11/19). In contrast, in AFT, only 29% (*n* = 13/42) of hospitalizations occurred within the first 5 weeks of treatment. Additionally, 50% (*n* = 8/16) of hospitalized participants in AFT had multiple admissions, whereas 29% (*n* = 5/17) in SyFT and 25% (*n* = 5/20) in FBT had multiple admissions.

## Discussion

These results support our first exploratory hypothesis that weight gain attributable to medical hospitalization would have little impact on EOT weight restoration, regardless of outpatient treatment type. This is an important finding because it supports the view that outpatient treatment is the most salient factor in overall weight restoration for adolescent AN. This is not to suggest that medical hospitalization is not necessary for the safe treatment of adolescent AN, but rather to recognize that the effects of such treatments on weight restoration are circumscribed. These results are aligned with prior literature (3), reporting medical stabilization alone when followed by FBT was as effective and less costly than longer term hospitalization aimed at full weight restoration.

Consistent with our second exploratory hypothesis predicting differing impacts of hospitalization according to treatment type, the data show that hospitalization weight gain had the greatest benefit for those treated using AFT relative to SyFT and FBT. AFT relies on helping the adolescent with AN learn to manage their eating and weight gain as opposed to parental management, thus hospitalization is likely to be needed more often to mitigate health risks as the adolescents themselves are learning to make necessary behavioral changes.

There are important limitations to consider. This secondary data analysis is exploratory in design, intended to generate hypotheses for further examination rather than conduct significance testing. The sample size, especially for AFT, is limited because the data is not available; to address the disproportionate sample sizes between groups, non-parametric statistics are reported. Although differences in hospitalized and non-hospitalized participants were not apparent at baseline, the data support existing literature that hospitalization is sometimes necessary to stabilize adolescents for outpatient care. For this subset of patients, it is possible that hospital admissions helped correct the course of treatment by allowing patients to stabilize vital signs and resume outpatient therapy. The outcome assessed is only related to weight restoration at EOT; while predictive of overall recovery at follow-up, it remains a limited outcome variable (6). Hospitalization in this study was restricted to medical necessity and the findings should not be extended to the use of psychiatric or behavioral hospitalization for adolescent AN or to other countries, health systems, or programs that use hospitalization for different indications in AN. There was a slight difference in treatment length and duration in the FBT treatment studies (24 sessions over 12 months vs. 16 sessions over 9 months), but this difference likely had little impact on hospital use, as almost all hospitalizations in FBT occurred early in treatment regardless of overall number of sessions or duration of treatment. While medical hospitalization criteria were consistent across sites and treatments, these criteria have not been systematically examined to determine their validity and reliability in preventing or limiting the medical sequelae of adolescent AN. Further, not all participants were admitted to the same medical hospitalization program; thus, it is possible that inpatient programming may differ across hospitalization sites and lead to differences in rate of weight gain. Across the seven sites, average hospitalization length of stay ranged from M = 7.6, SD = 2.1 to M = 21, SD = 2.The study's small sample size limited further investigation into site-related differences. The study's small sample size limited further investigation into site-related differences. Additionally, while all participants in this study were hospitalized consistent with published medical guidelines, we do not have data specific to the circumstances which preceded each hospitalization. Sex differences between treatment groups did not reach statistical significance (*p* = 0.14); however, it is possible that the distribution of sex across groups may have differentially impacted weight outcomes. Lastly, no hospitalized participants identified as American Indian or Alaska Native, and Native Hawaiian or Other Pacific Islander, which may limit generalizability of findings to these minority groups.

The results of this exploratory study suggest that further investigation of the role of medical hospitalization for adolescent AN is warranted. Additionally, clinicians providing AFT, FBT, and SyFT should be confident that these outpatient approaches, though varying in overall rates of recovery, do not obtain their respective treatment effects based on weight restoration as a result of medical hospitalization.

## Data Availability Statement

The raw data supporting the conclusions of this article will be made available by the authors, without undue reservation.

## Ethics Statement

The studies involving human participants were reviewed and approved by the appropriate Institutional Review Boards at each institution. Written informed consent to participate in this study was provided by the participants' legal guardian/next of kin.

## Author Contributions

ND: formal analysis, conceptualization, and writing. BM: conceptualization, writing, review, and editing. DL: funding acquisition (R01-MH-070620), writing, review, and editing. HB: funding acquisition (MH 076254), writing, review, and editing. BW: funding acquisition (MH 076252), writing, review, and editing. KH: funding acquisition (MH 076251), writing, review, and editing. DW: funding acquisition (MH 076255), writing, review, and editing. JL: funding acquisition (R01-MH-070621), supervision, conceptualization, and writing. All authors contributed to the article and approved the submitted version.

## Conflict of Interest

JL is co-director of the Training Institute for Child and Adolescent Eating Disorders, LLC, and received royalties from Routledge Press, Guilford Press, APA Press, and Oxford Press. DL receives royalties from Guilford Press and Routledge and is co-director of the Training Institute for Child and Adolescent Eating Disorders, LLC. The remaining authors declare that the research was conducted in the absence of any commercial or financial relationships that could be construed as a potential conflict of interest.
